# A supportive climate and low strain promote well-being and sustainable working life in the operation theatre

**DOI:** 10.1080/03009734.2018.1483451

**Published:** 2018-08-07

**Authors:** Robert Wålinder, Roma Runeson-Broberg, Erebouni Arakelian, Tobias Nordqvist, Andreas Runeson, Anna Rask-Andersen

**Affiliations:** a Occupational and Environmental Medicine, Department of Medical Sciences, Uppsala University, Uppsala, Sweden;; b Department of Surgical Sciences, Uppsala University, Uppsala, Sweden

**Keywords:** Anaesthetist, job demand-control-support model, hospital, nurse, occupational, operating room, psychosocial, zest for work

## Abstract

**Background:** Shortage of health-care workers e.g. in operating theatres is a global problem. A shortage of staff negatively affects patient outcomes, making it important to keep the employees from quitting. The aim of this survey was to study if well-being, zest for work, and thoughts about leaving work in an operating theatre can be related to the psychosocial work environment, as described by the job demand-control-support (JDCS) model.

**Methods:** A questionnaire was provided to personnel in operating theatres of seven Swedish hospitals (*n* = 1405, with a response rate of 68%) that included the JDCS model, personal factors, work ability, well-being, zest for work, and thoughts about leaving their position. Ordinal scale regression was used for analyses.

**Results:** A majority reported moderate to high zest for work (76%). A minority (30%) had sometimes thought during at least one month in the last year of leaving their position. Lower social support scores and high demands together with low control (high-strain) scores were related to lower well-being, lower zest for work, and more thoughts about leaving the position. Anaesthetists scored in the low-strain field, nurse anaesthetists and assistant nurses in the passive field, and operating nurses in the active field, in comparison to all personnel.

**Conclusion:** According to the JDCS model, both lower social support and high strain were related to lower well-being and negative thoughts about the position. Social support scores were about the same for different occupational groups in the operating theatre, and no occupation scored on average in the high-strain field.

## Introduction

Shortage of health-care workers is a global problem (1–3). Increasing demands of education and skills naturally also put more focus on work conditions. Health care involves more risk factors than most other areas of work, including: stress, threats and violence, shift-work, ergonomic strain, infectious diseases, radiation from X-ray and radionuclides, anaesthetic gases, cytostatics, and other chemical exposures. Nursing is also low paid, has low status, often involves bad working schedules, and sometimes insufficient management and support functions. Perhaps not surprisingly, highly educated and skilled health-care workers seek other jobs (4). There is also a professional debate about whether the paradigm of New Public Management that focuses on economic steering results in a trend of increasing demands, and less control by the employee over working conditions. On the individual level, changing jobs had a positive effect on employees with respect to job perception and job satisfaction and led to reduced fatigue and need for recovery (5).

The job demand-control-social support model (JDCS) has been widely used since it was introduced in 1979. It initially comprised the demands and control dimensions (6) before being supplemented with the dimension of support (7). According to this model, nurses and physicians were in the so-called ‘active field’, which was considered preferable to the ‘high-strain quadrant’ with both high demands and little control over the working situation (8). The JDCS model applied in health care has shown that job strain (defined as high psychological demands and low possibility of decision-making) was associated with hypertension (7,9), musculoskeletal symptoms among intensive care unit nurses (10), and somatic complaints among physicians (11).

Stress from high work demands is regarded as buffered when both decision authority and social support are high. In operating theatre settings, work overload, lack of job control, workplace atmosphere, and communication difficulties may cause stress at work, for example among anaesthetists (12,13). On the other hand, a review article claims that the most valuable factors leading to job satisfaction are worker autonomy, having control of the working environment, recognition of the worker’s value, professional relationships, leadership, and organizational justice (14). In addition to pay and promotion, supervisory satisfaction was inversely related to the intention to quit among nurse anaesthetists (15). In a study conducted on Finnish anaesthetists, low support from superiors and co-workers and low job control correlated with the intention to leave the job (16).

The aim of this study was to investigate if the psychosocial work environment, assessed via the job demand-control-support model, is related to well-being, zest for work, and intention to leave for personnel in the operating theatre.

## Methods

### Study population

The study population consisted of all workers in the operating theatre, except the surgeons, in seven hospitals in middle and northern Sweden—two university hospitals, four county hospitals, and one smaller hospital. A total of 1405 hospital workers were identified by the human resources departments. The local ethical committee approved the study (Dnr 2014/218).

### Questionnaire

The Swedish Job Demand Control Support Questionnaire (8), together with questions of personal characteristics (age, gender, occupation, and workplace), well-being, work ability (17,18), and zest for work (19) was sent to the study population by regular mail, with a response rate of 68%, after two reminders.

### Job demand-control-support (JDCS) model

**Figure 1.**
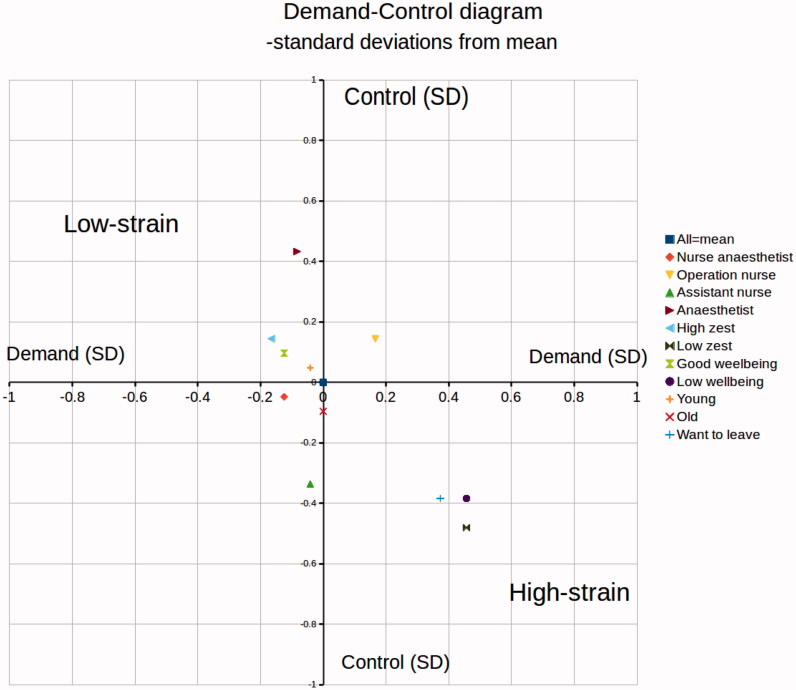
The two-dimensional demand-control diagram with the mean value of demands and control for the whole study population (*n* = 930) as reference value in origo, and mean ratings for the different subcategories relative to the group mean/origo (within ±1 standard deviation). The subcategories are nurse anaesthetist, operating nurse, assistant nurse, anaesthetist, high zest (‘I am happy about my job’, ‘I am rather happy about my job’, and ‘I am indifferent about my job’), low zest (‘I am somewhat uneasy about my job’ and ‘I have a strong aversion towards my job’), good well-being (above median), low well-being (below median), young (<30 y), old (>50 y), want to leave work (thoughts of leaving job: ‘Sometimes during one month’, ‘Sometimes during one week’, and ‘Every day’).

**Figure 2.**
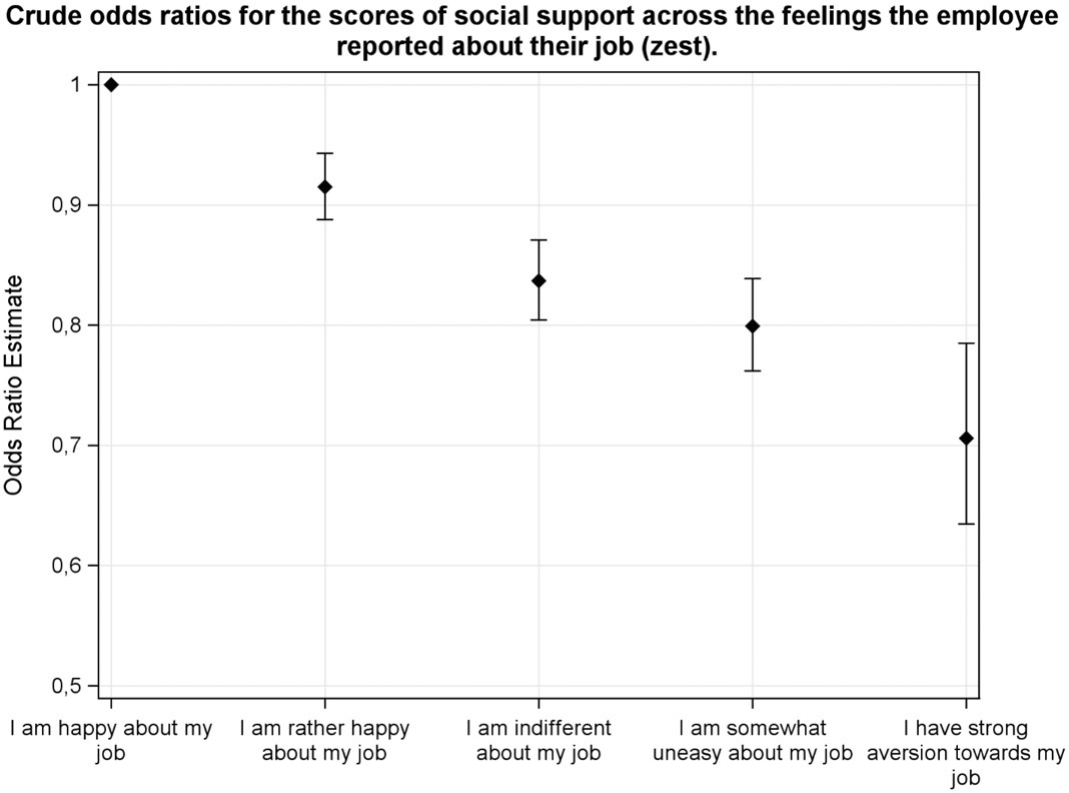
The support ratings are lower with less positive and more negative feelings about work, by ordinal regression (odds ratios with 95% confidence limits) for the study group. The alternative ‘I am happy about my job’ is used as reference category for the other four alternatives: ‘I am rather happy about my job’, ‘I am indifferent about my job’, ‘I am somewhat uneasy about my job’, and ‘I have a strong aversion towards my job’.

**Figure 3.**
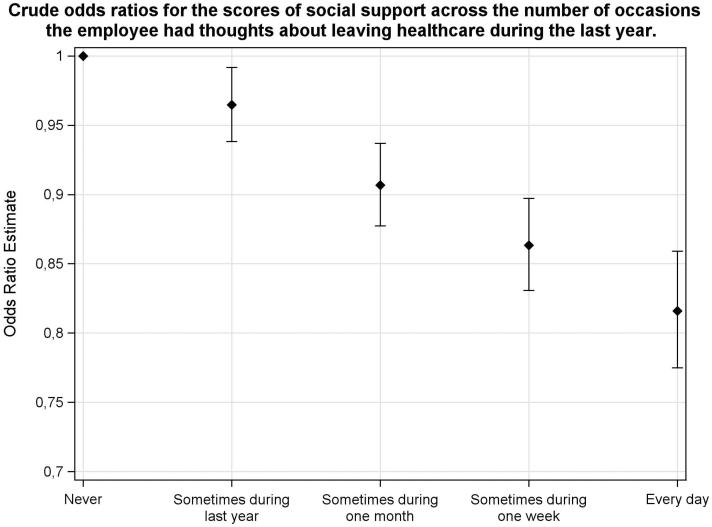
The support ratings are lower with higher frequency of thoughts about leaving work in health care, by ordinal regression (odds ratios with 95% confidence limits) for the study group. The lowest frequency of thoughts about leaving job in health care, ‘never during last month’, was used as reference for the other alternatives ‘Sometimes during one month’, ‘Sometimes during one week’, and ‘Every day.

**Figure 4.**
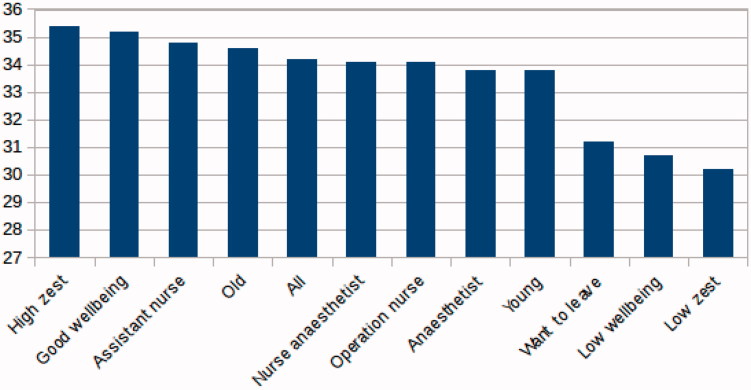
Ratings of social support across the categories (from high support to low support): high zest (‘I am happy about my job’, ‘I am rather happy about my job’, and ‘I am indifferent about my job’), good well-being (above median), assistant nurse, old (>50 y), all, nurse anaesthetist, operating nurse, anaesthetist, young (<30 y), want to leave (thoughts of leaving job: ‘Sometimes during one month’, ‘Sometimes during one week’, and ‘Every day’), low well-being (below median), low zest (‘I am somewhat uneasy about my job’ and ‘I have a strong aversion towards my job’).

**Figure 5.**
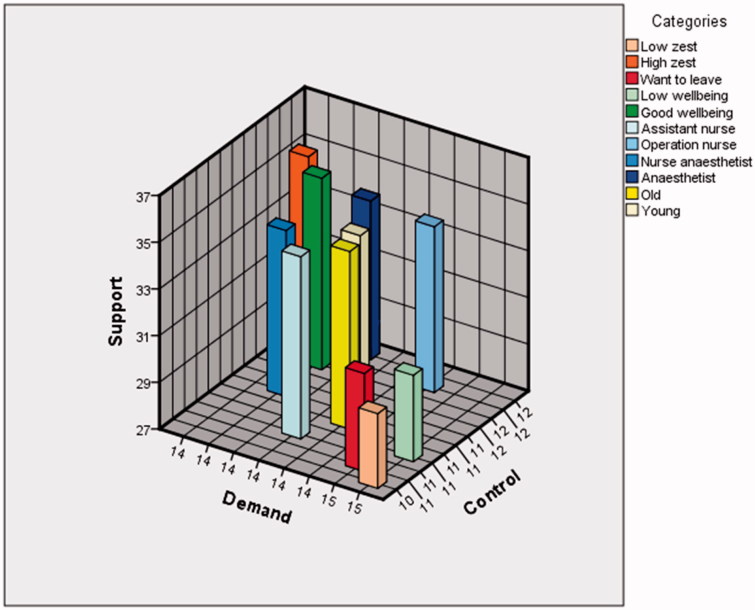
A three-dimensional demand-control-support model for the categories: low zest (‘I am somewhat uneasy about my job’ and ‘I have a strong aversion towards my job’), high zest (‘I am happy about my job’, ‘I am rather happy about my job’, and ‘I am indifferent about my job’), want to leave (thoughts of leaving job: ‘Sometimes during one month’, ‘Sometimes during one week’, and ‘Every day’), low well-being (below median), good well-being (above median), assistant nurse, operating nurse, nurse anaesthetist, anaesthetist, old (>50 y), and young (<30 y).

The Swedish Job Demand Control Support Questionnaire contains indices of demands in combination with control and support to assess the psychosocial work environment (6–8). The demands dimension consists of five questions, the control dimension of six questions, and the social support dimension of 16 questions, each with four score levels (1–4 points). In Table 1 the median values are used for the current study population sample as cut-off points giving the following ranges: demands with a possible range of 5–20 (high demands defined as scoring 14–20 and low demands as 5–13); control, 6–24 (high scoring 13–24, and low scoring 6–12); and support, 16–64 (high social support scoring 16–29, and low social support scoring 30–64). The social support dimension was divided into: manager support with a range of 5–20 (high defined as 5–8, and low 9–20), and colleague support with a range of 4–13 (high defined as 4–6, and low 7–13). In Figures 1, 4, and 5 the graphical presentations of different subcategories have been based on mean value ratings of the JDC scores. Since the JDC ratings are ordinal scales, the odds ratios of Table 2 and Figures 2 and 3 have been calculated by ordinal logistic regression.

**Table 1.** Absolute frequencies and percentages of personal characteristics and answers to the questionnaire for the study group.

	Nurse anaesthetist *n* = 312 (%)	Operating room nurse *n* = 266 (%)	Assistant nurse *n* = 240 (%)	Anaesthesiologist *n* = 112 (%)	Total *n* = 930 (%)
Age <30 y	27 (9)	18 (7)	17 (7)	4 (4)	66 (7)
Age 30–39 y	80 (26)	44 (16)	37 (16)	38 (34)	199 (22)
Age 40–49 y	92 (30)	82 (31)	42 (18)	28 (25)	244 (26)
Age ≥50 y	109 (35)	122 (46)	143 (60)	42 (38)	416 (45)
Mean age	45	48	50	46	47
Female gender	234 (76)	253 (95)	225 (94)	42 (38)	754 (82)
Reduced work ability (score 0–3/10)	10 (3)	7 (3)	12 (5)	4 (4)	33 (4)
Acceptable–good work ability (score 4–10/10)	298 (97)	259 (97)	227 (95)	108 (96)	892 (96)
Low demands^a^	144 (47)	98 (37)	108 (45)	50 (45)	400 (43)
High demands^a^	164 (53)	168 (63)	131 (55)	62 (55)	525 (57)
Low control^a^	159 (52)	111 (42)	155 (65)	31 (28)	456 (49)
High control^a^	149 (48)	155 (58)	84 (35)	81 (72)	469 (51)
Low peer support^a^	132 (43)	99 (37)	84 (35)	50 (45)	365 (39)
High peer support^a^	176 (57)	167 (63)	155 (65)	62 (55)	560 (61)
Low management support^a^	124 (40)	112 (42)	93 (39)	50 (45)	379 (41)
High management support^a^	184 (60)	154 (58)	146 (61)	62 (55)	546 (59)
Poor zest^b^	22 (7)	26 (10)	15 (6)	9 (8)	72 (8)
Neutral or good zest^c^	286 (93)	240 (90)	224 (94)	103 (92)	853 (92)
Wishing to leave^d^	113 (37)	80 (30)	63 (26)	23 (21)	279 (30)
Not wishing to leave^e^	193 (63)	187 (70)	178 (74)	88 (79)	646 (70)

a Dichotomously divided below and above the median.

b ‘I am somewhat uneasy about my job’ and ‘I have a strong aversion towards my job’.

c ‘I am happy about my job’, ‘I am rather happy about my job’, and ‘I am indifferent about my job’.

d Thought of leaving job: ‘Sometimes during one month’, ‘Sometimes during one week’, and ‘Every day’.

e Thought of leaving job: ‘Never’ and ‘Sometimes during last year’.

**Table 2.** The support ratings are lower with less positive and more negative feelings (zest) about work, and support ratings are also lower with more frequent thoughts about leaving health-care work, by ordinal regression (OR with 95% confidence limits) for the study group.

Feelings (zest) about work	Crude OR	Adjusted OR^a^
‘I am happy about my job’ (reference category)	1.0	1.0
I a I ‘I am rather happy about my job’	0.91 (0.89–0.94)	0.95 (0.91–0.98)
‘I am indifferent about my job’	0.84 (0.80–0.87)	0.88 (0.84–0.92)
‘I am somewhat uneasy about my job’	0.80 (0.76–0.84)	0.85 (0.80–0.90)
‘I have a strong aversion towards my job’	0.71 (0.64–0.78)	0.65 (0.51–0.83)
Thoughts of leaving job		
‘Never’ (reference category)	1.0	1.0
‘Sometimes during last year’	0.96 (0.94–0.99)	0.98 (0.95–1.005)
‘Sometimes during one month’	0.91 (0.88–0.94)	0.93 (0.90–0.97)
‘Sometimes during one week’	0.86 (0.83–0.90)	0.89 (0.85–0.93)
‘Every day’	0.82 (0.78–0.86)	0.86 (0.80–0.91)

a Adjusted for age, gender, occupation, and score of well-being.

### Work ability

A modified short version (17) of the Work Ability Index (WAI) scale (18) was used for the assessment of work ability. The self-rated ability to work was measured with a single question ‘How would you rate your ability to work today?’ using a 10-point scale, ranging from 0 (‘not being able to work right now’) to 10 (‘work ability now is best ever’).

### Well-being

Well-being was assessed by five questions, each with six levels: i) ‘Never’; ii) ‘A small portion of the time’; iii) ‘Less than half of the time’; iv) ‘More than half of the time’; v) ‘Most of the time’; vi) ‘Always’. The questions were: ‘How did you feel during the last two weeks?’: 1) ‘I was happy and in a good mood’; 2) ‘I was calm and relaxed’; 3) ‘I felt active and in good shape’; 4) ‘When I woke up I felt refreshed and rested’; 5) ‘My daily life was filled with interesting things’. The total score ranged from 0 to 25.

### Zest for work

Emotions about work on the way to work is called zest for work in the present study and has been assessed by one overall question: ‘How do you usually feel when you are on your way to work?’ The response option was to choose one of the following alternatives: i) ‘I feel happy and satisfied at the thought of the work that awaits me’; ii) ‘I have a quite positive feeling for work’; iii) ‘Neither positive nor negative feelings about work’; iv) ‘Feelings of some discomfort towards work’; and v) ‘Feelings of strong aversion towards work’. Answers were divided into high (alternative i or ii) or low (iv or v) zest groups, or for ordinal scale (i–v) analysis. This question was taken from a psychosocial questionnaire developed by Sigvard Rubenowitz and has been used earlier (19,20).

### Intention to stop working with health care

The intention to leave the health care sector was assessed with one question: ‘How often during the last year have you thought about giving up working with health care?’, with the following alternatives: i) ‘Never’; ii) ‘Sometimes during the year’; iii) ‘Sometimes during one month’; iv) ‘Sometimes during one week’; v) ‘Every day’. This question was taken and modified from the locked-in concept (21).

### Statistical methods

Absolute and relative frequencies above or below the group median values of questionnaire ratings for different subcategories have been given descriptively in Table 1. A graphical presentation of the different subcategories has been given in Figures 1, 4, and 5, based on mean score ratings. Ordinal logistic regression was applied to the outcomes zest for work, intention to leave, and work ability in Table 2 and Figures 2 and 3. Linear regression was applied to the relation of well-being. For the adjustment of age, occupation, sex, and well-being (in the ordinal logistic regression) these variables were included in the adjusted models. Two-way and three-way interactions in the models for social support were not statistically significant and are excluded from the results. Linear regression (GLIMMIX procedure) was applied to the relation of well-being. The residuals for the model were normally distributed, and there were no abnormal influential observations. Internal consistency of the variables in the JDCS model was tested by means of Cronbach’s alpha. Data analysis was performed by PROC LOGISTIC and PROC GLIMMIX in SAS 9.4.

## Results

Descriptive results (Table 1) show that a majority reported good or high work ability (96%) and moderate to high zest for work (76%), and a minority (30%) sometimes had thoughts about leaving their job during at least one month during the last year. Overall, there was very little variation (less than ±1 score point, or ±0.5 standard deviation) in mean scores between the different subcategories: nurse anaesthetist, operating nurse, assistant nurse, anaesthetist, high zest (‘I am happy about my job’, ‘I am rather happy about my job’, and ‘I am indifferent about my job’), low zest (‘I am somewhat uneasy about my job’ and ‘I have a strong aversion towards my job’), good well-being (above median), low well-being (below median), young (<30 y), old (>50 y), want to leave work (thoughts of leaving job: ‘Sometimes during one month’, ‘Sometimes during one week’, and ‘Every day’). These subcategories are graphically presented in the two-dimensional demand-control diagram (Figure 1) and the three-dimension job demand-control-support diagram (Figure 5). Three subcategories, those reporting ‘low well-being’, ‘low zest for work’, and a ‘high intention to leave work’, scored on average in the high-strain field (high demands and low control) compared to all personnel (Figure 1) and had lowest mean scores of support (Figures 4 and 5). No occupational subcategory was placed in the high-strain sector. Operating room nurses were the only category with mean scores in the active field (high demands and high control). The mean scores of nurse anaesthetists and assistant nurses were in the passive field (low demands and low control). The anaesthesiologists, younger employees, and those with good well-being and high zest for work were in the low-strain field (low demands and high control) (Figure 1).

Lower social support was related to lower scores of zest for work (Table 2; Figure 2) and more thoughts about leaving work (Table 2; Figure 3). Also, well-being was positively related to support ratings (*p* < 0.001), whereas work ability was not related to the support variable. Adjustment for age, gender, and occupation in an adjusted model did not change the crude relationships (Table 2). There were no statistically significant differences between the different occupational groups for the support variable (mean score differences <1). The support variable was related to the demands and control variables. Higher support was related to lower strain (high demands and low control) (OR 0.91, with 95% CI 0.89–0.93), lower demands (OR 0.89, with 95% CI 0.87–0.91), and higher control (OR 1.05, with 95% CI 1.03–1.07).

Test of consistency using Cronbach’s alpha showed high consistency for the demands and support variables, 0.76 and 0.81, respectively, but was poor for the control variable (0.5). An examination of two-way and three-way interactions showed positive interaction between well-being, zest for work, and less intention to leave.

## Discussion

Applying the job demand-control-support (JDCS) model among hospital workers in the operating theatre showed that social support and low strain were associated with workers’ well-being, zest for work, and higher intention to remain in the health-care sector. On the other hand, subjects who wanted to leave the health-care sector experienced lower support and high strain (high demands and low control), as did subjects who had low zest for work and low well-being. Also, other studies have shown that support or low strain in the JDCS model were related to well-being and job satisfaction among nurses and physicians (11,22–24). The cross-sectional design of the present study does not permit any causal conclusions to be made between well-being and positive thinking on the one hand and a supportive work climate or lower stress on the other (25). It is also possible that the workers are both products and producers of their work environment, and not just passive victims of environmental influences (26). Furthermore, since all data are self-reported the results might also be affected by recall bias, where healthy employees may experience less stress and have more positive thinking about the job and the social climate. On the other hand, there was no relation between work ability and the support variable.

Descriptively, a majority of the employees had positive thoughts about their jobs and did not plan to quit, but a smaller cluster of employees could be identified in the high-strain field of the job demand-control diagram reporting lower well-being, poor zest for work, and higher intention to leave. The same cluster also reported lower support, indicating a relation between work dissatisfaction and plans to leave in combination with a feeling of lower support and high strain.

A buffering effect of social support on high demands has been shown (27) and is also supported by the results of the present study. In a recent study, work over-commitment was related to poor zest for work (20). This is in agreement with the results of the present study if the positive relations between support and the indices of well-being, zest for work, and not wanting to leave work are interpreted as a positive buffering effect of support on employees’ health and work. Support was also prominent in a previous qualitative study of 22 informants from the same study group where the interviews pointed at recurring opinions about the importance of support, both from co-workers and managers (28).

Other studies have shown that the JDC model ‘job demand’ had a significant but negative relationship with job satisfaction, social support had a significant and positive relationship with job satisfaction, and high demands and lower social support affected the intention to leave (29,30). Also in the present study this relationship, often referred to as iso-strain, was established.

Dutch nurse anaesthetists and nurses in acute care units had less job satisfaction than anaesthetists, and the latter group also had a lower intention to leave their work than the nurses (31). Nurses from acute care units were in the strain quadrant of the JDCS model. Conversely, other nurses were commonly located in the ‘active’ quadrant. Independent of acute care settings, the highest level of education was associated with the highest job strain and the lowest level of control (32).

In the present study, Cronbach’s alpha tests of internal consistency were high for the questions about demands and support but poor for the control index. The validity of the questionnaire was originally tested with high validity and internal consistency for the demands index (33). But the index for control was only found to be well suited for population studies involving a wide range of work tasks. It is therefore not surprising that the limited range of work tasks in the operating theatre of the present study appears to make the questions of the control dimension less reliable. The response rate, 68%, is relatively high. Since limited information about the study population was available, apart from names, an analysis of the non-responders could not be carried out.

Since the origin of the demand-control-support model, new concepts of both lean and new public management have been introduced in Swedish hospitals that might influence the relevance of some of the questions in this study. The salary is a pertinent factor in the competition for competent professional labour. However, questions on salaries were not collected in this study, which calls for caution in interpretation of the responses of the present study.

Since only hospital workers in the operating theatre were included in the present study, a comparison with other workplaces could not be performed regarding the different fields of the demand-control diagram. Traditionally, nurses and physicians were placed in the so-called active field (8). This field of the diagram is considered preferable to the high-strain field, which has both high demands and little control over the working situation. In the present study, no occupation scored on average in the high-strain field, with the whole study group as reference.

In conclusion, most of the personnel in the operating theatre reported high zest for work, but one-third also had thought of leaving health care during at least one month the previous year. High social support and low strain were related to well-being, zest for work, and fewer thoughts about leaving their jobs. The cross-sectional design and possible self-reporting bias of the present study do not enable conclusions to be made about causal relationships, but results support previous studies showing a connection between support and lower stress on one hand, and workers’ well-being and sustainable working life on the other.
